# Establishing Age- and Sex-Specific Reference Intervals for Thyroid Function Tests in the Older People of Eastern Anatolia: A Population-Based Indirect Approach

**DOI:** 10.3390/medicina62030425

**Published:** 2026-02-24

**Authors:** Zekiye Çatak, Harun Fener, Hakan Ayyıldız, Zeynep Şimal Çokgüler

**Affiliations:** Department of Medical Biochemistry of Elazığ Fethi Sekin City Hospital, Health Sciences University, 23040 Elazığ, Türkiye

**Keywords:** thyroid function tests, reference intervals, aging, thyrotropin, free triiodothyronine, indirect method, geriatrics, Türkiye

## Abstract

*Background and Objectives:* Given that hormone levels vary with age, the application of age-specific reference intervals in older populations is clinically essential. In this study, we aimed to establish age- and sex-specific reference intervals (RIs) for serum free triiodothyronine (fT3), free thyroxine (fT4), and thyroid-stimulating hormone (TSH) in healthy individuals aged ≥65 in Eastern Turkey using an indirect statistical method. *Materials and Methods:* This retrospective study included 3835 individuals (1986 males and 1849 females) who were evaluated at Elazığ Fethi Sekin City Hospital between 2020 and 2025. According to the Clinical and Laboratory Standards Institute (CLSI) C28-A3 guidelines, reference intervals were determined using a laboratory database–based indirect reference interval estimation approach with nonparametric percentile methods following a posteriori reference population selection, and the Harris–Boyd criteria were applied for age and sex partitioning. *Results:* The established reference intervals for those aged ≥65 years were 2.40–4.03 pg/mL for fT3, 0.60–1.27 ng/dL for fT4, and 0.41–3.94 mIU/L for TSH. While fT3 levels declined with age, TSH and fT4 levels did not differ consistently across age subgroups. Sex-based differences were significant: fT3 levels were higher in males, whereas fT4 and TSH levels were higher in females. According to the Harris–Boyd analysis, separate reference intervals are recommended for males and females. *Conclusions:* For healthy older individuals living in Eastern Türkiye, sex-specific reference intervals should be used for thyroid function tests, whereas age-specific reference intervals are sufficient for fT3.

## 1. Introduction

Thyroid hormones play a critical role in the regulation of the cardiovascular, neurological, metabolic, and musculoskeletal systems; therefore, accurate assessment of serum free triiodothyronine (fT3), free thyroxine (fT4), and thyroid-stimulating hormone (TSH) levels is of clinical importance in the diagnosis and monitoring of thyroid dysfunction [1]. However, the reference intervals (RIs) used to interpret these parameters must reflect the statistical distribution and the physiological alterations associated with aging. The aging process is characterized by reduced sensitivity within the hypothalamic–pituitary–thyroid (HPT) axis, perturbations in peripheral deiodinase-mediated conversion, and diminished end-organ responsiveness to circulating hormones [2,3]. Given these homeostatic changes, thyroid profiles should be carefully interpreted to distinguish normal aging from thyroid disease.

Previous studies indicate that age, sex, and ethnic variations remarkably impact thyroid hormone levels, suggesting that a single RI may not be suitable for all populations [4,5]. Furthermore, iodine intake, as well as genetic and environmental factors, contribute to variability in RIs between different populations [5,6]. In Türkiye, thyroid RIs for those aged ≥65 years are mostly based on secondary analyses from studies with wider age ranges. Therefore, comprehensive data focused specifically on the older people remains limited [7,8]. Accordingly, it is necessary to establish local RIs to ensure more reliable diagnoses in geriatric care. The rapidly aging demographic structure of Türkiye further underscores the importance of accurate diagnosis and treatment of thyroid diseases in older adults, making the determination of age- and sex-specific RIs even more critical [9,10]. Indirect statistical approaches enable the retrospective analysis of large laboratory information system databases, providing RIs that more accurately reflect population-specific biological distributions. Utilizing indirect statistical methods, as endorsed by the Clinical and Laboratory Standards Institute (CLSI) EP28-A3c guideline, provides a fast, cost-effective, and reliable alternative, particularly for older populations, where recruiting healthy reference individuals can be challenging [11,12].

The objective of this study was to address a notable gap in the literature on thyroid function tests in older adults by establishing age- and sex-specific RIs for fT3, fT4, and TSH in healthy individuals aged ≥65 years residing in Eastern Türkiye using an indirect statistical method, with the aim of improving the diagnostic precision and clinical interpretation of thyroid function in geriatric practice.

## 2. Materials and Methods

**Figure 1:**
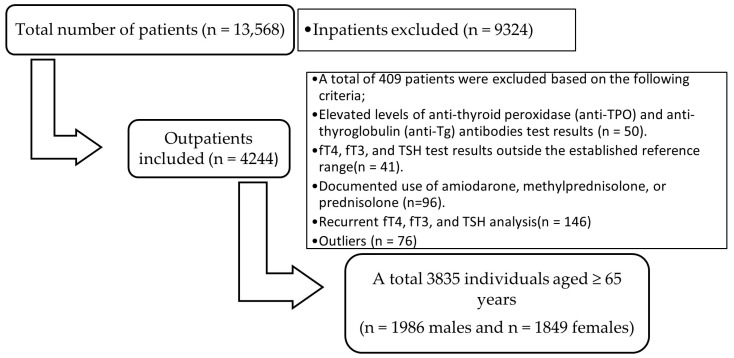
Flowchart illustrating patient selection and exclusion process for reference interval determination.

The study protocol was approved by the Ethics Committee of Elazığ Fethi Sekin City Hospital, University of Health Sciences (No. 2025/20-5), and all procedures were performed in accordance with the Declaration of Helsinki. For this study, TSH, fT4, and fT3 test results of patients aged ≥65 years who were evaluated at Elazığ Fethi Sekin City Hospital for general health examinations between 1 January 2020, and 1 December 2025, were retrospectively obtained from the Laboratory Information System of the Central Biochemistry Laboratory. Of the approximately 4244 outpatients who underwent all three analyses simultaneously, several groups were excluded from the study: those with positive anti-thyroid peroxidase (anti-TPO) and anti-thyroglobulin (anti-Tg) antibody results (n = 50); patients with abnormal thyroid function test results, defined as fT4, fT3, or TSH levels outside the laboratory-established RIs (n = 41); those documented using amiodarone, methylprednisolone, or prednisolone (n = 96); and patients with duplicate results (n = 146). Anti-TPO and anti-Tg testing was not routinely performed in all individuals and was evaluated only when available. After exclusion of extreme values using the Tukey interquartile range (IQR) method in accordance with the CLSI C28-A3 guidelines (n = 76), 3835 individuals aged ≥65 years (1986 males and 1849 females) were included in the study (Figure 1). All analyses were performed using fasting morning blood samples.

All measurements were performed using a UniCel DxI 800 analyzer (Beckman Coulter Access Immunoassay Systems, Brea, CA, USA). The limits of detection were 0.003 μIU/mL for TSH, 0.22 ng/dL for fT4, and 1.1 pg/mL for fT3. The TSH assay had a functional sensitivity of 0.02 μIU/mL, at a coefficient of variation (CV) of 10%, and was classified as a third-generation TSH assay. According to the manufacturer’s specifications, the intra- and inter-assay CVs for all parameters were within acceptable limits. In our laboratory, two levels of internal quality control (low and high) were analyzed daily. The CV values for TSH, fT3, and fT4 met the performance standards. In addition, all assays were regularly evaluated within the external quality assessment program conducted by the Turkish Association of Clinical Biochemistry Specialists (KBUDEK), which confirmed satisfactory analytical performance. The reference ranges used in our laboratory were 2.6–4.37 pg/mL for fT3, 0.34–5.33 mIU/L for TSH, and 0.6–1.16 ng/dL for fT4. For comparison, the manufacturer’s adult RIs were 0.38–5.33 mIU/L for TSH, 2.5–3.9 pg/mL for fT3, and 0.61–1.12 ng/dL for fT4.

Statistical analyses were performed using SPSS version 21.0 (IBM Corp., Armonk, NY, USA). The normality of the variables was assessed using the Kolmogorov–Smirnov and Shapiro–Wilk tests. Because the data did not follow a normal distribution, the RIs were calculated using the median and the 2.5th–97.5th percentiles. RIs were determined using a nonparametric method according to the CLSI and Laboratory Standards Institute C28-A3 guidelines. Outliers were identified and excluded using Tukey’s IQR method. Values below Q1 − 1.5 × IQR or above Q3 + 1.5 × IQR were excluded. Subsequently, reference limits were established based on an outlier-free dataset [13]. The 90% bootstrap confidence intervals for the lower and upper reference limits were calculated using 5000 resamples. The Harris–Boyd method was applied to assess whether differences between age groups 65–74 years (n = 2577), 75–84 years (n = 1054), and ≥85 years (n = 204) and between sexes warranted the establishment of separate reference intervals. Because the ≥85 male and female subgroups did not meet the CLSI-recommended minimum sample size for nonparametric reference interval estimation (n ≥ 120), formal subgroup-specific reference intervals were not established for these strata. During the decision-making process, the calculated *Z* values (*Z_calc_*) were compared with the critical *Z* values (*Z_crit_*). When *Z_calc_* exceeded *Z_crit_*, separate RIs were defined for the relevant age or sex. Accordingly, decisions regarding reference interval partitioning were based on *Z*-scores rather than *p*-values, and statistical significance refers to *Z_calc_* exceeding *Z*_crit_.

As this study was based on a retrospective laboratory database, direct clinical verification of individual health status (e.g., through physical examination or medical history) was not possible. Nevertheless, in line with the CLSI C28-A3 recommendations for indirect RI estimation, it was assumed that most biochemical profiles represented individuals without overt thyroid dysfunction. This assumption is inherent to indirect reference interval estimation and is widely accepted in large-scale laboratory studies. Advanced statistical mixture modeling techniques were not applied; instead, a database-based a posteriori reference population selection strategy followed by nonparametric percentile estimation was implemented in accordance with CLSI C28-A3 guidance. Comprehensive data cleaning procedures were implemented to ensure the accuracy of RIs, including the exclusion of individuals with positive thyroid antibodies, relevant medication use, duplicate entries, and statistically defined outliers (Figure 1). These steps were taken to minimize the influence of pathological values and to approximate a healthy older population. The resulting RIs were expected to improve the diagnostic precision of subclinical thyroid dysfunction by reducing the risk of misclassification associated with generalized adult ranges.

## 3. Results

**Figure 2:**
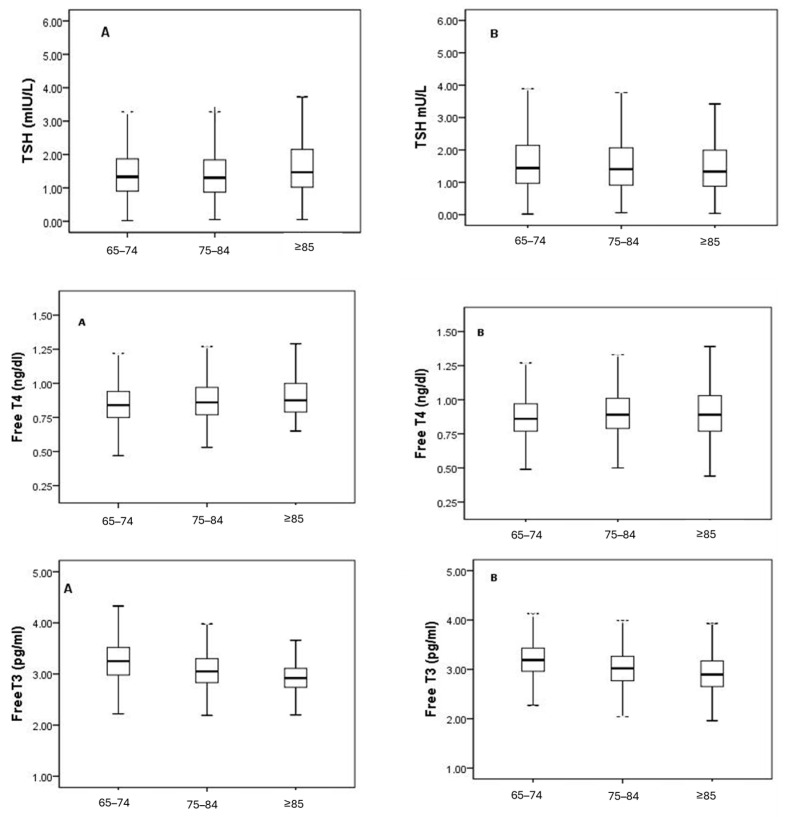
Distribution of serum free T3, free T4, and TSH levels across age groups in males and females. Panels A and B represent male and female individuals, respectively. Box plots represent the median and interquartile range; whiskers indicate the 2.5th–97.5th percentiles. Small dots represent outlier values.

In this study, fT3, fT4, and TSH levels of 3835 elderly individuals without known thyroid dysfunction were evaluated according to age and sex. In the overall population, the median (2.5th–97.5th percentile) values were 3.15 pg/mL (2.40–4.03) for fT3, 0.86 ng/dL (0.60–1.27) for fT4, and 1.36 mIU/L (0.41–3.94) for TSH (Table 1). When compared by sex, fT3 levels were higher in males (3.18 vs. 3.12 pg/mL), whereas fT4 and TSH levels were higher in females (Table 1). Harris–Boyd analysis indicated that the *Z*_calc_ values for fT3 (5.73), fT4 (6.12), and TSH (4.95) exceeded the *Z*_crit_ values. These findings indicate that separate RIs should be defined for males and females. When analyzing the age groups, a gradual decrease in fT3 levels was observed with increasing age (65–74 years: 3.22; 75–84 years: 3.03; ≥85 years: 2.91 pg/mL) (Table 2). The 90% bootstrap confidence intervals for the lower and upper reference limits are provided in Table 1 and Table 2. Figure 2 illustrates this pattern, showing a downward trend in fT3 levels in both males and females, while fT4 and TSH levels varied only slightly between age groups. According to the Harris–Boyd analysis (Table 3), differences in fT3 levels were observed between the 65–74 and 75–84 age groups and between the 65–74 and ≥85 age groups. No differences warranting reference interval partitioning were observed for fT4. For TSH, a difference was observed only between the 65–74 and 75–84 age groups, without a consistent age-related pattern (*Z*_calc_ = 4.78 > 3). This isolated statistical difference did not support clinically meaningful age-specific partitioning. When males and females were stratified by age (Table 4), differences in fT3 levels warranting partitioning were observed between the 65–74 and 75–84 age groups in both sexes (*Z*_calc_ > *Z*_crit_). In contrast, fT4 and TSH levels did not differ consistently across age groups for either sex (*Z*_calc_* < Z_crit_*). The 90% bootstrap confidence intervals for the age- and sex-specific reference limits are provided in Supplementary Table S1. Taken together, fT3 was the only parameter that varied consistently with age and sex, decreasing with advancing age and being slightly higher in males. fT4 and TSH levels were relatively more stable across subgroups. These results indicate that age- and sex-specific RIs should be used for fT3 levels, whereas for fT4 and TSH, sex-specific but age-independent reference intervals are sufficient (Figure 2).

## 4. Discussion

Accurate assessment of thyroid function in the geriatric population is crucial because of age-related changes in hormone levels. In this study, thyroid function test RIs were evaluated in individuals aged ≥65 years residing in Eastern Türkiye and compared with previously published data. Compared with previously published RI studies conducted in Türkiye, our findings showed overall concordance, with minor variations that may reflect differences in study populations and analytical methodologies [14].

In our study, we observed that fT3 levels clearly decreased with age (median 65–74 years: 3.22; 75–84 years: 3.03; ≥85 years: 2.91 pg/mL). This finding can be explained by an age-related decline in peripheral deiodinase activity and is consistent with previous literature [15,16]. Consequently, age-specific RIs for fT3 are more appropriate for older individuals. In contrast, fT4 levels did not differ significantly between the age groups. Accordingly, a common RI for fT4 is sufficient. This finding is consistent with those reported in previous studies [13,17,18].

When comparing our results with the manufacturer’s RIs, we found that the ranges for fT3 and fT4 were largely consistent with the manufacturer’s values (0.38–5.33 mIU/L, 2.5–3.9 pg/mL, and 0.61–1.12 ng/dL for TSH, fT3, and fT4, respectively). However, our upper reference limit for TSH (0.41–3.94 mIU/L) was lower than both the manufacturer’s RI and the values reported in multiple international studies. For example, the NHANES III data showed that the median TSH increased to 2.08 mIU/L in individuals aged ≥80 years [13]. Similarly, a study of the Chinese population demonstrated that for every 10-year increase in age, there was an increase of approximately 0.5 mIU/L in the 97.5th percentile of TSH [19]. In contrast, we found that TSH levels did not increase significantly with age. According to the Harris–Boyd analysis, only a limited difference was observed between the 65–74 and 75–84 age groups (*Z_calc_* > *Z_crit_*). This finding is consistent with similar studies conducted in Türkiye. For instance, previous studies have reported that TSH levels do not increase with age in individuals older than 50 years. Instead, in some subgroups, both the lower and upper percentile values for TSH may shift toward lower values compared with those observed in the general population [7,8,18]. Nevertheless, this finding should be interpreted with caution, as the relatively small number of individuals aged ≥85 years may have limited the robustness of age-specific estimates in very advanced age.

TSH dynamics during aging vary considerably across different populations. A comprehensive review by Zhai et al. (2024) [17] reported that changes in thyrotropin levels in older populations did not follow a uniformly increasing pattern. Instead, the iodine intake status, the prevalence of autoimmunity, ethnic and environmental factors, and methodological differences were identified as the main determinants of this heterogeneity [17]. Effective iodine replacement programs implemented in Türkiye for many years may be an important factor in limiting the age-related increase in TSH levels. Atmış et al. (2021) reported that adequate iodine levels can help stabilize the age-related increase in TSH levels [20].

In addition to environmental, ethnic, and methodological factors, sex is an important determinant of thyroid hormone distribution and the establishment of RIs. Accordingly, when analyzed by sex, we identified TSH RIs of 0.43–3.67 mIU/L in males and 0.40–4.19 mIU/L in females. Large population-based studies have also reported higher TSH levels in females than in males, and this difference may be related to sex-specific variations in feedback mechanisms within the HPT axis [13,21]. In our study, fT4 levels were higher in females (median fT4: 0.87 vs. 0.85), whereas fT3 levels were higher in males (median fT3: 3.18 vs. 3.12 pg/mL). These findings may be explained by long-term estrogen exposure, which increases the synthesis of thyroid-binding globulin, thereby affecting free hormone fractions and leading to differences in the HPT feedback mechanism [15,22]. According to the Harris–Boyd criterion, *Z*_calc_ values exceeding *Z*_crit_ values for all three parameters (fT3, fT4, and TSH) justify the use of sex-specific RIs in older individuals. Although such sex-related differences have been reported, it is well recognized that the magnitude and direction of these differences may vary depending on population demographics, history of hormonal exposure, and methodological approaches used for RI determination [15,16,21].

However, when male and female participants were evaluated separately within age groups, these differences were less pronounced. Based on the Harris–Boyd criterion, a significant sex difference was identified for fT3 in the 65–74 and 75–84 age groups, and partitioning was recommended for these groups. In contrast, for fT4 and TSH, *Z*_calc_ values were lower than *Z_crit_* values in most age groups, indicating that age-based partitioning was not required. Accordingly, the effect of sex may be more pronounced on peripheral thyroid hormone metabolism and fT3 conversion.

This study has several limitations. First, because the study was conducted in a single geographic region, the generalizability of the findings to the entire older population in Türkiye may be limited. Second, because the RIs were determined by an indirect method, anti-TPO and anti-Tg testing, as well as thyroid ultrasonography, were not performed in all participants. Therefore, some structural or autoimmune thyroid disorders may not have been detected, and clinically silent thyroid diseases may not have been completely excluded. The presence of additional potential confounders affecting thyroid function in older adults—such as thyroid hormone replacement therapy, antithyroid drugs, lithium use, biotin supplementation, chronic kidney disease, or acute non-thyroidal illness—could not be systematically evaluated due to limitations of the retrospective laboratory database. To minimize the inclusion of individuals with overt thyroid dysfunction in the absence of complete clinical verification, results outside the existing laboratory reference intervals were excluded. While this strategy was methodologically pragmatic in a large retrospective dataset, it may have introduced a degree of circularity, potentially leading the newly derived reference intervals to resemble the pre-existing ones, narrowing the distribution tails and possibly influencing the estimated upper limits, particularly for TSH. However, the number of individuals excluded solely on the basis of values outside the existing laboratory reference intervals was relatively small compared with the total study population, suggesting that the overall impact on percentile-based estimation was likely limited. Although the study period overlapped with the COVID-19 pandemic, COVID-19-related thyroid function alterations are generally transient, and a residual effect cannot be completely excluded. In addition, the relatively low representation of individuals aged ≥85 years may have limited the robustness of the RI estimates and the ability to comprehensively evaluate TSH dynamics at a very advanced age. These limitations should be considered when interpreting the observed age-related decline in fT3 levels and the lack of a consistent age-dependent TSH pattern.

Despite these limitations, the indirect retrospective approach is widely used in real-world laboratory settings and is endorsed by CLSI C28-A3 for large-scale RI studies. Moreover, the use of increasingly sensitive TSH and thyroid antibody assays has enabled more accurate selection of reference populations, contributing to a gradual reduction in the upper reference limit of TSH over the past decade [23].

## 5. Conclusions

In conclusion, this study demonstrated that in elderly individuals without known thyroid dysfunction living in Eastern Türkiye, sex-specific RIs should be used for thyroid function tests, whereas age-specific RIs are sufficient only for fT3. Because the observed differences between the subgroups for TSH did not show a consistent age-dependent pattern, it was considered unnecessary to partition the RI. This approach is consistent with the CLSI C28-A3 recommendations, which emphasize that differences identified between subgroups according to the partitioning criteria do not automatically justify RI partitioning unless they demonstrate consistent, clinically relevant, and interpretable patterns. In addition, the absence of a consistent age-related increase in TSH levels suggests that TSH behavior in older adults may be more adaptive and influenced by ethnic, environmental, and individual factors. From a clinical perspective, the use of general adult RIs in this population may increase the risk of misclassification, particularly for subclinical thyroid dysfunction. These findings highlight the importance of population-specific RIs. Further studies with larger sample sizes and/or prospective designs are warranted to confirm these findings.

## Figures and Tables

**Table 1:** Median and 2.5th–97.5th percentile values of serum free T3, free T4, and TSH according to the overall population and sex.

Analyte	Overall (n = 3835)	Male (n = 1986)	Female (n = 1849)
Free T3 (pg/mL)	3.15 (2.40–4.03)	3.18 (2.43–4.05)	3.12 (2.37–3.99)
	[2.37–2.42; 3.99–4.05]	[2.41–2.47; 4.0–4.09]	[2.34–2.40; 3.94–4.05]
Free T4 (ng/dL)	0.86 (0.60–1.27)	0.85 (0.59–1.24)	0.87 (0.61–1.30)
	[0.59–0.61; 1.25–1.29]	[0.58–0.61; 1.21–1.27]	[0.6–0.63; 1.27–1.33]
TSH (mIU/L)	1.36 (0.41–3.94)	1.33 (0.42–3.67)	1.42 (0.40–4.18)
	[0.39–0.43; 3.83–4.15]	[0.4–0.45; 3.56–3.86]	[0.36–0.43; 4.02–4.3]

Values are presented as median (2.5th–97.5th percentiles). Brackets indicate 90% bootstrap confidence intervals for the lower (2.5th percentile) and upper (97.5th percentile) reference limits (5000 resamples). According to the Harris–Boyd analysis, the calculated *Z*-values (*Z_calc_*) for fT3(5.73), fT4(6.12), and TSH(4.95) exceeded the critical threshold (*Z_crit_* = 3.0) between the sexes.

**Table 2:** Median and 2.5th–97.5th percentile values of serum free T3, free T4, and TSH according to age groups.

Analyte	65–74 Years (n = 2577)	75–84 Years (n = 1054)	≥85 (n = 204)
Free T3 (pg/mL)	3.22 (2.52–4.07)	3.03(2.32–3.90)	2.91 (2.17–3.96)
	[2.47–2.56; 4.03–4.09]	[2.21–2.36; 3.82–3.98]	[2.03–2.22; 3.84–4.19]
Free T4 (ng/dL)	0.85 (0.61–1.22)	0.88(0.59–1.32)	0.88 (0.61–1.36)
	[0.59–0.62; 1.2–1.25]	[0.57–0.61; 1.29–1.37]	[0.55–0.65; 1.3–1.54]
TSH (mIU/L)	1.38 (0.43–4.03)	1.35(0.39–3.87)	1.37 (0.18–3.69)
	[0.4–0.44; 3.87–4.19]	[0.25–0.42; 3.62–4.21]	[0.05–0.47; 3.22–4.01]

Values are presented as median (2.5th–97.5th percentile). Brackets indicate 90% bootstrap confidence intervals for the lower (2.5th percentile) and upper (97.5th percentile) reference limits (5000 resamples).

**Table 3:** Partitioning decisions for thyroid hormone parameters by age based on the Harris–Boyd analysis.

	Comparison	*Z*_calc_	*Z*_crit_	Decision
Free T3	65–74 vs. 75–84	3.32	3	Partition
Free T3	65–74 vs. ≥85	4.07	3	Partition
Free T3	75–84 vs. ≥85	2.22	3	No
Free T4	65–74 vs. 75–84	2.62	3	No
Free T4	65–74 vs. ≥85	0.09	3	No
Free T4	75–84 vs. ≥85	0.95	3	No
TSH	65–74 vs. 75–84	4.78	3	Partition
TSH	65–74 vs. ≥85	1.2	3	No
TSH	75–84 vs. ≥85	1.2	3	No

**Table 4:** Median and 2.5th–97.5th percentile values of serum free T3, free T4, and TSH according to age and sex.

Analyte	65–74 Years		75–84 Years		≥85 Years	
	Male (n = 1382)	Female (n = 1195)	Male (n = 518)	Female (n = 536)	Male (n = 86)	Female (n = 118)
Free T3 (pg/mL)	3.25 (2.53–4.09)	3.19 (2.49–4.04)	3.05 (2.36–3.87)	3.02 (2.27–3.93)	2.92 (2.20–3.94)	2.90 (2.03–3.98)
Free T4 (ng/dL)	0.84 (0.59–1.20)	0.86 (0.63–1.25)	0.86 (0.58–1.29)	0.89 (0.59–1.36)	0.88 (0.67–1.50)	0.89 (0.55–1.37)
TSH (mIU/L)	1.33 (0.43–3.81)	1.44 (0.42–4.19)	1.31 (0.39–3.56)	1.41 (0.33–4.22)	1.47 (0.22–3.82)	1.33 (0.09–3.42)

Values are presented as median (2.5th–97.5th percentile).

## Data Availability

The original contributions of this study are included in this article. Further inquiries can be directed to the corresponding author.

## Supplementary Materials

The following supporting information can be downloaded at: https://www.mdpi.com/article/10.3390/medicina62030425/s1, Table S1: Age- and sex-specific reference intervals (2.5th–97.5th percentiles) with corresponding 90% bootstrap confidence intervals (5000 resamples).
