# Supplementary material for: Establishing Age- and Sex-Specific Reference Intervals for Thyroid Function Tests in the Older People of Eastern Anatolia: A Population-Based Indirect Approach
Source: Medicina (Kaunas). 2026 Feb 24;62(3):425. doi: 10.3390/medicina62030425 (PMC13027790; doi:10.3390/medicina62030425)
Supplement: Supplementary file 1 [file medicina-62-00425-s001.zip › medicina-4120930-supplementary.pdf]

Supplementary Table S1.
Age- and sex-specific reference intervals (2.5th–97.5th percentiles) with corresponding 90% bootstrap confidence intervals (5000 resamples).

Age Group	Sex (n)	Analyte	Lower RI	Upper RI	90% CI (Lower Limit)	90% CI (Upper Limit)
65–74	Male (1382)	Free T3	2.53	4.09	2.45–2.58	4.04–4.14
65–74	Male (1382)	Free T4	0.59	1.20	0.57–0.61	1.17–1.23
65–74	Male (1382)	TSH	0.43	3.81	0.40–0.47	3.58–4.08
65–74	Female (1195)	Free T3	2.49	4.04	2.44–2.56	3.96–4.08
65–74	Female (1195)	Free T4	0.63	1.25	0.61–0.64	1.21–1.27
65–74	Female (1195)	TSH	0.42	4.19	0.38–0.44	4.02–4.43
75–84	Male (518)	Free T3	2.36	3.87	2.26–2.43	3.81–4.04
75–84	Male (518)	Free T4	0.58	1.29	0.57–0.63	1.24–1.34
75–84	Male (518)	TSH	0.39	3.56	0.25–0.44	3.30–3.68
75–84	Female (536)	Free T3	2.27	3.93	2.13–2.34	3.73–3.99
75–84	Female (536)	Free T4	0.59	1.36	0.56–0.61	1.34–1.40
75–84	Female (536)	TSH	0.33	4.22	0.15–0.42	3.89–4.69
≥85	Male (86)	Free T3	2.20	3.94	2.09–2.32	3.75–4.17
≥85	Male (86)	Free T4	0.67	1.50	0.65–0.71	1.23–1.54
≥85	Male (86)	TSH	0.22	3.82	0.05–0.54	3.25–4.35
≥85	Female (118)	Free T3	2.03	3.98	1.96–2.22	3.73–4.36
≥85	Female (118)	Free T4	0.55	1.37	0.44–0.62	1.23–1.40
≥85	Female (118)	TSH	0.09	3.42	0.04–0.50	3.15–4.91

Values are presented as 2.5th–97.5th percentiles. Confidence intervals were calculated using bootstrap resampling (5000 iterations). Results for the ≥85 subgroup should be interpreted with caution due to smaller sample sizes.
